# Summer Excursions

**Published:** 1864-08

**Authors:** 


### HALL'S JOURNAL OF HEALTH.

Vol. XI. AUGUST, 1864. No. 8.

### SUMMER EXCURSIONS.

THE true method of renovating the health and invigorating the constitution is for gentlemen, scholars, clerks, and others of sedentary occupation, to travel on foot through mountainous, regions, camp out, fish, hunt, botanize, or with hammer and satchel read the "rocks" of every locality through which they pass. Ladies should eschew the luxurious carriage-trunks of warehouse size, and skirts forty yards in circumference, and in the old-time "riding habit" ride on horseback over the hills and far-away. A week thus spent is worth more than a month's sojourn at the spa or the sea-side, where the daily routine is but a succession of alternate loungings, gluttonies, late suppers, and midnight revelry. The best time is not during the sweltering heats and suffocating dusts of summer, but the cool and bracing autumn. The following description of a friend, of a ramble in the Green Mountains of Vermont is timely. Some with more leisure might spend a few months among the "Japs," or other peoples little known. Let us, then, "in the mind," take a stroll by the side of the "daffodil meadows," by the brink o[illegible]f the birchen-shadowed and rock-fretted stream, that "bathes the green woods in freshening spray-mist;" over the breezy mountains, redolent with the healthful perfume of the fir, the spr[illegible]ce, and the pine.

If you see[illegible] such scenes, gentle reader, go to Northern Vermont—"the Switzer[illegible]and of America," a region not much trodden by the footsteps of Cockney tourists and pleasure-seekers, but abounding in scenery of such variety as to meet the requisitions of every shade of taste and fancy; streams rushing, in "fury and foam," down the hills, and hurrying away through pleasant vallies, to join "*la belle rivière*," the beautiful Connecticut; lakelets, nameless, because numberless, reposing "serene as the Sabbath, and bright as a holy day," in the shadow of the hills; mountains of every grade and hue, rock-ribbed and forest-crowned, from those of humble name, up to that magnificent range from which the State appropriately receives its appellation.

Near the village of Brookford, in Orange county, is a singular conical eminence, standing, like "The Peak of Teneriffe, alone in solitary grandeur," called " Wright's Mountain." Allured by the description of the fine view from its summit, I undertook the ascent, in company with a friend, a task attended with some difficulty, owing to the fact that its sides are extremely precipitous, and the total absence of any path, at least, any that we could discover. Encouraged, however, by the extreme enthusiasm of my companion, who, leading the way, forcibly reminded me of the picture of Christian ascending the hill " Difficulty," on which I used to gaze with extreme delight, in an old copy of " Pilgrim's Progress " which I thumbed when a child; his untiring perseverance and exhaustless ingenuity in surmounting obstacles, in the form of enormous overhanging rocks,. every moment threatening ruin, like the stone of Tantalus, steep as the sides of a church; his free use of the motto " Excelsior," and assurances oft-repeated, that we were near the summit, that the desired eminence was at length gained; when, unlike most who climb such giddy heights, we were amply repaid for all our toil. On the South, and contiguous to the base of the mountain, flowed the sparkling waters of Wait's River, a monstrous misnomer, by the way, for a more rapid hurrying little spirit of a stream is nowhere to be found beyond the silver line of its waters, a landscape of great beauty and variety, terminated by the " purple rim " of the White Mountains.

On the east, the ever lovely Connecticut, dotted, as to its banks, with picturesque villages that are the exclusive glory of New England, beyond the " Franconia Range," prominent among these, " Moose Hillock," with its scarred sides and wooded back; far beyond, the ever-present glory of the famous Mount Washington; north, another variegated landscape, dotted with farms whose fields from this elevation appeared like the beds of a carefully cultivated garden; with the appropriately named " Blue Mountains " of Ryegate in the back ground; eastward, the " Orange Hills," compelled to assume this humble appellation in presence of their more dignified neighbors, the Green Mountains beyond.

Amid all this limitless variety of hill and valley, mountain and plain, straying streamlets and rolling rivers, my eye rested with peculiar interest upon one spot—a house, a barn, an orchard, a brook—that was *all!* these, but dimly distinguished at the distance of several miles, the home of my childhood, the parent nest from which I had wandered long years ago. That dim streak, is the road that my " baby footsteps " trod to the " village school;" there are the rocks where I climbed, the hills yet radiant with the dreams of youth; from the waters of that crystal brook, on a well-remembered evening, when dark storm-clouds were gathering, and muttering thunderings were heard in the distant west, with an enthusiasm that Christopher North himself might have envied, were the first, most beautiful, and prized of trout lured to their doom by the youngest of old Izaak Walton's disciples!—trivial things, but just such as the flood of years cannot efface, while they bear with them perhaps, much of higher importance. What wonder, if with these scenes mingled other forms, some of whom "remain until this present, but the greater part have fallen asleep." Nor, in their absence, can the coming years bring such joys as those of the past have borne away! But the *train!* what, of thought?—no, of *cars!* We must hurry down, only looking at "The Sybil's Cave," a wild, wierd place, amid rocks all tumbled about in admirable disorder, which Blair pronounces one of the elements of the *sublime;* in at one opening, through a dark window passage, up and out at another, suggestive of wolves and bears, who doubtless once revelled in these halls, long since abandoned for more remote retreats.

In the county of Orleans, the hills are not quite so steep or rugged as those of Orange, consequently the scenery is scarcely so striking and picturesque, although quite equal in views of both the great mountain ranges: indeed, through all this region, almost every turn of the road presents a landscape worthy of the pencil of a Turner, or the pen of a Ruskin. This portion of the State abounds in large "granite boulders," a species of which, the "spotted granite of Craftsbury," is peculiar to this region, and of great interest to the scientific. In the town of Westborough, on the farm of Mr. Waugh, is to be seen one of these huge boulders, twelve feet in length and nine in height, weighing, according to measurement, eighty tons, resting upon the outer edge of another of much greater dimensions, and so nicely poised that the effort of a single man is sufficient to move it through an arc of several inches, while the combined force of a large party with levers and other appliances of power, was insufficient to move it out of its place.

As I swayed the huge mass to and fro, with a single hand, I could not but wonder how it came there, and wish that, since there are said to be "sermons in stones," that this one had a tongue to tell its history perfectly, sure that a few words would throw more light upon the mystery of Creation, than all that geologists have ever written. The clerical friend who was with me, curious in the doctrine of chances, was soon immersed in the calculation of the number against this stone being found precisely in this situation! the solution was not reached when we parted. Another, weighing fifty tons, and in a similar position, stands near by, placed there by the same power; but whether by the throes of the same convulsion, who can tell?

Passing by many objects of beauty and interest, we come to the crowning glory of this region, "Willoughby Lake," a most romantic little tarn, reposing calmly in the embrace of two giant hills which rear their rugged heights two thousand feet upon either side —itself some five or six miles in length, from one to two in breadth— to the hill on the left, they have given the name "Mount Hor," to that upon the right, "Mount Pisgah."

We approached the upper or eastern end of the lake, just as the sun was setting behind Mount Hor, and shedding his last beams upon the summit of Pisgah; it required no great effort of the imagination to transform the distant landscape that was discovered at the foot of the vista formed by the mountains, into "the land that lies beyond the flood;" the dome-like dress of Owl's Hood, distinctly visible at the distance of forty miles, into one of the "everlasting hills;" this flood of light that was poured upon the sky, and bathed these mountain summits, into the "celestial glory" of that city that has no need of the sun, for there is no night there.

A night's rest in the very comfortable "Lake House," and a capital breakfast, of which the chief attraction was those incomparable trout which abound in the lake and the streams of the neighborhood, prepared me for the ascent of Pisgah, and the enjoyment of the scene from its summit.

A walk of some two miles, by no means excessively steep or difficult, conducts to the top of the mountain. If the view yields in magnificence to that which greeted the vision of Moses as his eye swept the glories of the promised inheritance from "the top of Pisgah that is over against Jericho," I can well conceive that his soul would be filled with sadness at the thought that he "could not enter in."

Mount Washington and his fellows, Cornel's Hump, Jay Peak, Nose and Chin, and Owl's Head, with the glassy surface of Memphremagog at his base, all in full view; the country spread out like a map before us far as the eye could reach, dotted with villages and sown with small lakes that flashed like transparent glass in the rays of the morning sun.

A descent of a few hundred feet brings to the extreme verge of a cliff that overhangs the lake, at an elevation of twelve hundred feet, and apparently so directly above, that a single step would measure the horizontal distance. Though not much inclined to enthusiasm, here I must confess myself overcome with mingled emotions of awe and sublimity. The immense height, the tremendous chasm, with the lake at the bottom, the wooded slopes and sides of Mount Hor, rising like a wall of emerald beyond a landscape such as I have attempted to describe, all combine to form one of the most sublime scenes, certainly, to be met with in the limits of our country.

In the evening, accompanied by two or three pleasant companions, we went out upon the lake for the purpose of hearing the famous Echo. When we had rowed between one and two miles, and were near the base of the left-hand mountain, our oarsman blew a loud blast with a shell; rolling in loud reverberations, until we distinctly counted the sixth repetition, it died away in the far distance. We repeated it again and again, with the same grand effect, until the chill air, and the moon rising in beauty and casting her silver radiance upon the transparent water, warned us that it was the hour to return. Those who have heard "Green Mountain echoes," by Dodsworth's Band, may form some idea of this magnificent echo, of which this piece of music is a successful imitation.

There are many other things of which I would speak, did I not fear that this article is already prolonged to a degree which may render it unsuitable for your pages.

I returned from my rambles in Vermont, regretting that I could not have remained where I found so much to delight and attract; and heartily recommend the region, at which we have but glanced, to all who are seeking for health or enjoyment in the more quiet and picturesque scenes of Nature.

